# DRL-Based Scheduling for AoI Minimization in CR Networks with Perfect Sensing

**DOI:** 10.3390/e27080855

**Published:** 2025-08-11

**Authors:** Juan Sun, Shubin Zhang, Xinjie Yu

**Affiliations:** 1 School of Computer and Data Engineering, NingboTech University, Ningbo 315104, China; sunjuan@nbt.edu.cn; 2 Computer Science and Technology, Zhejiang University of Technology, Hangzhou 310023, China; zhangshubin@zjut.edu.cn

**Keywords:** Age of Information, RF energy harvesting, CRN, dynamic programming, deep Q-network

## Abstract

Age of Information (AoI) is a newly introduced metric that quantifies the freshness and timeliness of data, playing a crucial role in applications reliant on time-sensitive information. Minimizing AoI through optimal scheduling is challenging, especially in energy-constrained Internet of Things (IoT) networks. In this work, we begin by analyzing a simplified cognitive radio network (CRN) where a single secondary user (SU) harvests RF energy from the primary user and transmits status update packets when the PU spectrum is available. Time is divided into equal time slots, and the SU performs either energy harvesting, spectrum sensing, or status update transmission in each slot. To optimize the AoI within the CRN, we formulate the sequential decision-making process as a partially observable Markov decision process (POMDP) and employ dynamic programming to determine optimal actions. Then, we extend our investigation to evaluate the long-term average weighted sum of AoIs for a multi-SU CRN. Unlike the single-SU scenario, decisions must be made regarding which SU performs sensing and which SU forwards the status update packs. Given the partially observable nature of the PU spectrum, we propose an enhanced Deep Q-Network (DQN) algorithm. Simulation results demonstrate that the proposed policies significantly outperform the myopic policy. Additionally, we analyze the effect of various parameter settings on system performance.

## 1. Introduction

Over the past few decades, the burgeoning number of wireless devices and the rising need for wide-band services have caused a considerable depletion of the available licensed spectrum. A report by the Federal Communications Commission (FCC) reveals that the utilization rate of the licensed spectrum is below 10 percent at any specific time and location [1]. This significant spectrum underutilization has spurred the development of cognitive radio (CR), a technology designed to enable secondary users (SUs) to access primary users’ (PUs) licensed bands while guaranteeing quality of service (QoS) for PUs [2,3,4,5]. In cognitive radio networks (CRNs), two primary spectrum sharing strategies are commonly employed: underlay and overlay. In the underlay approach, both the primary and secondary networks simultaneously transmit data over the licensed spectrum, with interference from the secondary network constrained to a predefined threshold to ensure the protection of the primary network [6]. On the other hand, the overlay strategy allows the secondary network to transmit data only when the primary network is not using the spectrum. This approach protects the primary network by enabling the secondary network to dynamically detect an idle spectrum and opportunistically access it without causing interference [4].

Beyond the issue of spectrum scarcity, energy constraints also present a substantial challenge for the evolution of future wireless networks. Recently, the ability to scavenge energy from radio frequency (RF) signals has been recognized as a promising avenue for powering low-power wireless devices. By using RF energy-harvesting technology, wireless powered communication networks (WPCNs) offer a solution to the unpredictability and intermittency of traditional charging methods by harnessing energy from renewable sources [7,8,9,10,11]. With widespread attention to WPCNs, we have previously conducted some related work. In [12], we address the problem of maximizing the aggregate computation rate in a WPCN comprising multiple hybrid access points (HAPs) and IoT devices, where we introduce a deep reinforcement learning (DRL) algorithm for determining near-optimal offloading strategies and develop an efficient Lagrangian duality-based approach to derive the corresponding optimal time allocation. In [13], we investigate the application of physical layer security (PLS) as a mechanism for ensuring the privacy of the secondary network’s communication, where both the SU and jammer can harvest RF energy from the primary network transmissions. In [14], we focus on the problem of jointly optimizing the duration of wireless power transmission, the allocation of transmission time for individual edge devices, and the decision regarding partial task offloading to achieve the maximum sum computation rate.

To address the aforementioned challenges of spectrum scarcity and energy deficiency, RF energy harvesting in CRNs has gained significant attention. This approach enhances both energy and spectral efficiency, enabling battery-free SUs to simultaneously capture energy and spectra from PUs [15]. Considering an SU intermittently generating real-time messages with a delivery deadline, the authors in [16] derive the probability of the timely delivery of data packs.

In contrast to traditional CR systems, where the SU maintains time-slot synchronization with the PU, the authors in [17] explore an RF energy-harvesting CRN with an unslotted PU, focusing on sensing intervals to strike a balance between spectrum access and energy harvesting. However, refs. [16,17] examine simple CRNs with a single primary link and a single secondary link. On the other hand, refs. [18,19,20] investigate scenarios involving multiple SUs or multiple PUs. In [18], a multichannel selection strategy is proposed to maximize the average throughput of SUs in RF energy-harvesting CRNs. In [19], the authors focus on a hybrid energy-harvesting SU model, where the SU harvests energy from both solar and ambient RF signals. A convex framework is developed to maximize throughput by optimizing the sensing duration, active probability, and detection threshold for each SU. In [20], multi-hop transmission with a time division multiple access (TDMA) echanism is employed by the SUs, where the authors address throughput maximization by jointly optimizing transmission power and time.

Existing research primarily focuses on the throughput and delay performance of SUs while considering the QoS requirements of PUs in RF energy-harvesting CRNs [16,17,18,19,20]. However, many emerging CRN applications, such as smart buildings, vehicle-to-vehicle networking, environmental monitoring, and health monitoring [21,22,23], demand the timely delivery of physical process status updates. For example, in environmental monitoring systems, sensor nodes must frequently collect and transmit critical parameters (e.g., temperature, humidity) to ensure accurate real-time tracking, highlighting the necessity of low-latency status updates. Age of Information (AoI), a newly proposed network performance metric, can be used to characterize the timeliness of status updates in RF energy-harvesting CRNs. The AoI metric tracks the temporal freshness of received status updates. It measures the duration from the generation of the latest successfully received update to the current time at the destination [24,25,26,27].

There have been some initial studies on the AoI of CRNs [28,29,30,31,32,33]. Ref. [28] investigates an interference-free interweave CR system, developing an optimized framing and scheduling approach that maximizes energy efficiency while adhering to AoI constraints. In [29], the authors conduct a comprehensive analysis of the average peak AoI, deriving asymptotic closed-form expressions for both underlay and overlay transmission schemes under ideal spectrum sensing conditions. The authors in [30] investigate an overlay CR system where SUs may either transmit their own data or serve as relays for PU transmissions. The study develops a constrained Markov decision process framework (CMDP) to optimize the joint status update and relaying strategy, simultaneously addressing AoI minimization and energy efficiency. The authors in [31] study AoI minimization for energy-harvesting SU, where the SU can harvest energy from ambient energy sources. The optimal sensing and update decision problems are initially formulated as partially observable Markov decision process (POMDP) frameworks and then solved using dynamic programming. Following [31], the authors in [32] investigate AoI minimization for wireless energy-harvesting SUs in a CRN where multiple SUs and multiple PUs coexist. In each time slot, the SUs either harvest energy from the PU transmissions or deliver status updates when no primary receivers are present in the associated guard zone. The authors first derive the outage probability of the PUs and then propose a greedy policy for the SUs performing status updates. Considering the difficulty for PUs and SUs to achieve perfect time slot synchronization, the authors in [33] study the AoI minimization problem in a CRN with unslotted PUs. The authors derived the closed-form expression for the average AoI by conducting a Markov chain analysis.

To the best of our knowledge, relatively less work has been conducted on AoI minimization in RF energy-harvesting CRNs. Motivated by this, this work focuses on optimizing the average AoI for an SU powered by RF energy harvesting from PU’s transmissions within a defined time frame. The optimization process rigorously considers both the energy causality constraint and limitations imposed by spectrum availability. In this system model, the SU relies on energy scavenged from the PU to first perform spectrum sensing. Subsequently, if the PU vacates the spectrum, the SU further decides whether to transmit its status update data packet to the common base station (CBS). Additionally, we consider a broader scenario where multiple SUs, powered by the PU, are deployed to monitor various physical processes and send their status update data to a shared CBS. The goal is to minimize the long-term average weighted sum of AoI (sum-AoI), which represents the total of AoI values for each SU at the CBS. This research presents a significant advancement in the study of RF energy-harvesting CRNs by, for the first time, simultaneously exploring AoI minimization in both single-SU and multiple-SU scenarios. Furthermore, we innovatively employ a DQN approach to solve the POMDP problem, where only the PU spectrum belief is known.

It is worth noting that our paper significantly differs from [31,32,33,34,35]. In [31], the SUs harvest energy from the ambient environment, and the scenario of multiple SUs is not considered. In contrast, our problem setup involves SUs harvesting energy from PU transmissions, and we consider the presence of multiple SUs. Furthermore, sensing actions are not considered in [32], whereas we incorporate them. The scenario considered in [33] also differs from ours. In [33], the PU’s activity is characterized by its ability to randomly seize the channel and potentially interrupt ongoing SU transmissions mid-slot, leading to SU transmission failures. However, we consider PUs and SUs achieving perfect time slot synchronization. Finally, refs. [31,32,33] do not utilize DRL-based methods, while our approach is based on DRL. Refs. [34,35] use the DDPG algorithm within an overlay CRN, while we employ the DQN. The main contributions of this paper are summarized as follows.

- We focus on minimizing the average AoI for a single RF energy-harvesting SU with a fixed time duration. The SU obtains its energy supply through harvesting energy from PU transmissions and is enabled to deliver status update data packets to the CBS only when the PU spectrum is identified as being in an idle condition. In each discrete time interval, the SU’s spectrum sensing and status update decisions are adaptively made considering its current energy reserves, AoI, channel link quality, and the availability of the PU spectrum. The decision-making problem under consideration is modeled using a POMDP with discrete state and action sets. The optimal policy for this model is then derived through the application of dynamic programming.
- We extend the scenario to multiple SUs, where the objective is to minimize the long-term average weighted sum-AoI by making adaptive sensing and update decisions. We model this decision-making problem as a POMDP with finite state and action spaces. However, due to the computational challenges posed by the extreme curse of dimensionality in the state space of the POMDP, we propose an improved DQN approach to learn the optimal policy. This enhanced DQN approach is tailored to handle the POMDP problem, where the partially observable state is modeled as a Markov chain.
- We validate through extensive simulations that the proposed policies essentially improve the system performance compared to the myopic policy, and we also analyze the impact of system parameter settings on the system performance.

The remaining part of this paper is organized as follows. Section 2 describes the system model for the RF energy-harvesting CRN with one SU. Section 3 formulates the finite-horizon AoI minimization problem for the single-SU scenario as a POMDP framework and solves it through dynamic programming. Section 4 presents the system model for RF energy-harvesting CRN with multiple SUs. Section 5 formulates the infinite-horizon AoI minimization problem for the multiple-SU scenario as a POMDP framework and solves it using a new DQN approach. Section 6 presents simulation results. Finally, Section 7 concludes the paper.

## 2. System Model for RF Energy-Harvesting CRN with One SU

**Figure 1:**
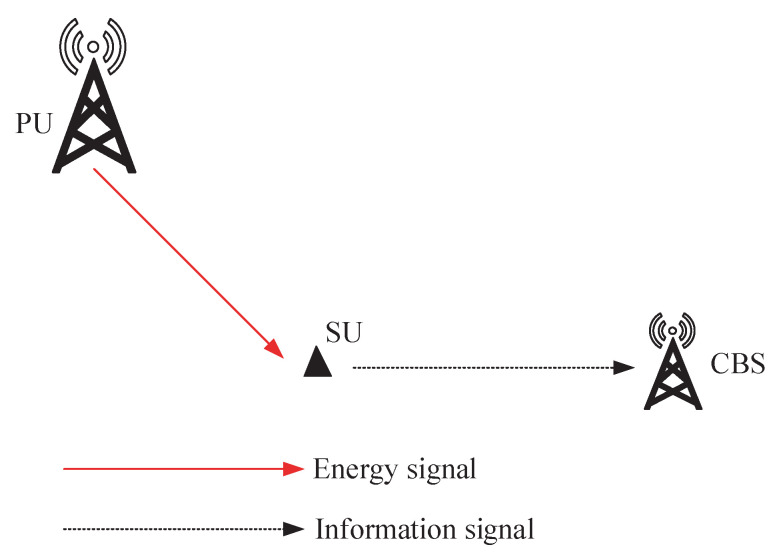
System model for one SU. In each time slot, the SU can harvest energy from PU transmissions and deliver the status update data pack to the CBS when the channel is in an idle state.

Figure 1 illustrates the CRN under investigation, which comprises one SU, one PU, and a CBS receiving status update packs sent by the SU. The SU, equipped with a sensor, monitors a physical process and transmits status updates regarding its observations to the CBS. Lacking an internal power supply, it operates by scavenging energy from the PU’s transmissions. Additionally, spectrum access is granted to it when the PU vacates the channel, enabling opportunistic operation. We adopt a discrete-time framework, dividing the operational period into *T* time slots, indexed by t=0,1,…,T−1. To simplify the analysis, we assume a unit duration of one second for each time slot.

### 2.1. Primary User Model

The PU is granted preferential access to the spectrum, and its channel occupancy dynamics are characterized by a two-state Markov chain, encompassing active (A) and idle (I) states [36,37]. During each time slot, the PU alternates between data transmission in the active state and silence in the idle state. We define the transition probabilities of the Markov chain as $p_{ii}$ and $p_{ai}$, with $p_{ii}$ indicating the persistence of the idle state and $p_{ai}$ quantifying the likelihood of a transition from the active to the idle state. For t=0,1,…,T−1, we have

(1) $$p_{ii}=P\left(q_{t+1}=I|q_{t}=I\right),$$

(2) $$p_{ai}=P\left(q_{t+1}=I|q_{t}=A\right).$$

The SU possesses prior knowledge of the transition probabilities, acquired through extended measurement periods.

### 2.2. Secondary User Model

The SU keeps time-slotted synchronization with the PU. The SU deliberates whether to sense the spectrum at the start of each time slot. Should the SU opt not to perform spectrum sensing, the entirety of the time slot is dedicated to energy harvesting from the PU’s transmissions, a process that is exclusively enabled during the PU’s active state and ceases when the PU is idle. Should the SU opt to perform spectrum sensing, a predetermined fraction of the time slot is assigned to this task. We assume the perfect sensing outcome for the SU [31,33]. Our decision to assume perfect sensing in this work was primarily driven by the need to isolate and focus on the core contributions of our proposed DQN algorithm regarding AoI minimization. This ideal scenario allows us to establish a performance upper bound and evaluate the fundamental effectiveness of our DQN framework without the added complexity introduced by sensing errors. If the spectrum is sensed to be occupied by the PU, the SU harvests energy in the rest time of this time slot; otherwise, the SU needs to further decide whether to update when the sensing action ends. The SU aims to minimize the average AoI by employing optimal spectrum sensing and status update policies throughout the entire operational period. The action executed within the time slot *t* is designated as $x_{t}=\left(\theta_{t},\phi_{t}\right)$. $\theta_{t}\in \left\{0\left(\mathrm{not}\;\mathrm{sense}\right),1\left(\mathrm{sense}\right)\right\}$, which denotes spectrum sensing performed by the SU, while $\phi_{t}\in \left\{0\left(\mathrm{not}\;\mathrm{update}\right),1\left(\mathrm{update}\right)\right\}$ signifies the SU’s status update operation. The SU’s decisions are influenced by its state and the statistical knowledge of the PU’s activity, as described in the following.

*(1) Belief model:* The SU observes the availability of the PU’s spectrum by adaptively detecting and opportunistically accessing the idle spectrum. Leveraging the SU’s action and observation history, the belief state concerning the PU’s spectrum activity is formulated. Specifically, at the commencement of each time slot, the SU constructs the belief state, denoted as $\varrho_{t}$, which represents the conditional probability of the PU being in the idle state, given the SU’s historical actions and observations.

*(2) Channel model:* The channel power gains, denoted by $h_{t}$ (SU-to-CBS) and $g_{t}$  (PU-to-SU) for the time slot *t*, are modeled as independent and identically distributed (i.i.d.) random variables over time. The channels are modeled using the Rayleigh fading model and are assumed to experience quasi-static flat fading. That is, they exhibit temporal stability within individual time slots but undergo variations across successive time slots. Adhering to a well-established practice within the wireless communication community, the channel power gains during the current time slot are assumed to be perfectly known [38].

*(3) Energy-harvesting model:* The SU can harvest energy from the PU transmissions. For the SU, harvest-then-transmit protocol is employed [39]. The SU initiates energy harvesting from PU transmissions, subsequently employing the acquired energy to transmit status update packets to the CBS. In general, there are two scenarios for energy harvesting: (1) when a non-sensing decision coincides with the primary user’s active transmission and (2) following a sensing operation that confirms the PU’s occupancy. Let τ represent the time allocated for energy harvesting, η the energy conversion efficiency, and *P* the PU’s transmission power. The amount of energy acquired by the SU through harvesting is expressed as

(3) $$E_{H,m}^{t}=\tau \eta Pg_{t},\;\;t=0,\;1,\;\ldots ,\;T-1,\;m=1,\;2.$$

The two distinct values of *m* correspond to the two scenarios for energy harvesting. The harvested energy is used for both spectrum sensing operations and the subsequent transmission of status update packets via the wireless channel. We define δ and $\tau_{s}$ as the energy and time used for sensing, respectively. Meanwhile, we define $E_{T}^{t}$ as the energy consumption and $\tau_{t}$ as the time required for data transmission within the time slot *t*. Let $\sigma^{2}$ and *W* represent the noise power at the CBS and the bandwidth, respectively. The noise is modeled as Gaussian white noise. According to Shannon’s formula, $E_{T}^{t}$ is expressed as

(4) $$E_{T}^{t}=\frac{\sigma^{2}\tau_{t}}{h_{t}}\left(2^{\frac{S}{\tau_{t}W}}-1\right),$$

The SU’s battery capacity is $B_{\max}$. We define $b_{t}$  as the battery’s state of charge during the time slot *t*, which evolves as

(5) $$b_{t+1}=\min\left\{b_{t}+E_{H,m}^{t}-\theta_{t}\delta -\phi_{t}E_{T}^{t},B_{\max}\right\},\;\;t=0,\;1,\ldots ,\;T-1.$$

Here, $E_{H,m}^{t}$ is the energy captured by the SU, $\theta_{t}\delta$ the energy consumed for spectrum sensing, and $\phi_{t}E_{T}^{t}$ the energy consumed for data transmission, all within the time slot *t*. It is worth noting that if the PU spectrum is occupied, the SU performs energy harvesting, and the battery state increases by $E_{H,m}^{t}$. Conversely, if the PU spectrum is idle and the SU decides to send a status update pack, the battery state decreases by $\phi_{t}E_{T}^{t}$. Therefore, the energy causality constraint is ensured by

(6) $$\theta_{t}\delta +\phi_{t}E_{T}^{t}\leq b_{t},\;\;t=0,\;1,\;2,\;\ldots ,\;T-1.$$

*(4) Age of information:* Let $a_{t}$ represent the AoI in the time slot *t*. The upper bound of the AoI is denoted as $A_{\max}$, calculated as $A_{\max}=a_{0}+T$, where $a_{t}\in A≜\left\{1,\;2,\;\ldots ,\;A_{\max}\right\}$. For every time slot, where an update decision is made by the SU, one status update packet is generated and dispatched, utilizing the generate-at-will scheme [28,29,30,31,32,33]. The size of data packet *S* is small enough that it can be generated and updated immediately after the update decision is made and received by the end of the time slot. Following the successful reception of the update at the CBS, the AoI is reset to 1; conversely, it increases by 1. AoI evolves over time slots and is given by

(7) $$a_{t+1}=\left\{\begin{array}{c} 1,\;\;\;\;\;\mathrm{if}\;\;x_{t}=\left(1,1\right),\\ a_{t}+1,\;\;\;\;\;\;\;\mathrm{otherwise}. \end{array}\right.$$

Equation (7) posits an error-free channel, which ensures the successful reception of status update data packets at the CBS upon an update decision. The average AoI across *T* time slots is computed as

(8) $$\bar{A}=\frac{1}{T}\sum_{t=0}^{T-1}a_{t},\;\;t=0,\;1,\;2,\;\ldots ,\;T-1.$$

## 3. FINITE POMDP FORMULATION for RF Energy-Harvesting CRN with One SU

### 3.1. POMDP Formulation

To address the SU’s AoI minimization problem, the optimal sensing and update strategies are modeled using a POMDP. The elements of this POMDP are detailed below.

- *Actions:* Initially, the SU determines whether to perform spectrum sensing. If it does not sense the spectrum, then it harvests energy from the PU transmissions and does not deliver the status update data pack, i.e., $x_{t}=\left(0,0\right)$. If it senses the spectrum and finds that the spectrum is occupied by the PU, it also cannot perform an update i.e., $x_{t}=\left(1,0\right)$. If it senses the spectrum and finds that the spectrum is vacated by the PU, it needs to further decide whether to deliver the status update data pack based on its AoI, channel state from it to the CBS, the channel state from the PU to it, and the energy availability, i.e., $x_{t}=\left(1,0\right)$ or $x_{t}=\left(1,1\right)$. Consequently, the action in each time slot can be defined as $x_{t}=\left(\theta_{t},\phi_{t}\right)\in X≜\left\{\left(0,0\right),\left(1,0\right),\left(1,1\right):b_{t}\geq \theta_{t}\delta +\phi_{t}E_{T}^{t}\right\}$, where $\theta_{t}\in \Gamma_{\theta }≜\left\{0,1:b_{t}\geq \theta_{t}\delta \right\}$ and $\phi_{t}\in \Gamma_{\phi }≜\left\{0,1:b_{t}\geq \delta +\phi_{t}E_{T}^{t}\right\}$.
- *Observations and beliefs:* The PU’s state is observed as ${\hat{q}}_{t}\in \left\{A,I\right\}$, while the belief $\varrho_{t}\in \left[0,1\right]$ signifies the probability of spectrum availability. This belief is dynamically updated based on the sequence of past actions and observations, according to the transition function $\varrho_{t+1}=\Lambda \left(\varrho_{t+1}\right)$, as follows:
  (9) $$\varrho_{t+1}=\left\{\begin{array}{c} \Lambda_{0}\left(\varrho_{t}\right)=\varrho_{t}p_{ii}+\left(1-\varrho_{t}\right)p_{ai},\;\mathrm{if}\;\;\theta_{t}=0,\;b_{t+1}=b_{t},\\ \Lambda_{A}\left(\varrho_{t}\right)=p_{ai},\;\;\;\;\;\;\;\;\;\;\;\mathrm{if}\;\;\left(\theta_{t}=0,\;b_{t+1}\neq b_{t}\right),\\ \;\;\;\;\;\;\;\;\;\;\;\;\;\;\;\;\;\;\;\;\;\;\mathrm{or}\;(\theta_{t}=1,\;{\hat{q}}_{t}=A),\\ \Lambda_{I}\left(\varrho_{t}\right)=p_{ii},\;\;\;\;\;\;\;\;\;\;\;\;\;\;\mathrm{if}\;\;\theta_{t}=1,\;{\hat{q}}_{t}=I. \end{array}\right.$$
  Specifically, if the SU chooses not to perform spectrum sensing, the subsequent belief update is contingent upon two possible scenarios: (1) If the battery state remains unchanged, the belief is updated based solely on the PU state Markov chain. (2) If the battery energy increases, it means that the PU channel in the time slot *t* is busy, and $\varrho_{t+1}=p_{ai}$. When the SU performs spectrum sensing, the outcome of this process reflects the actual occupancy status of the spectrum. Equation (9) reveals that the SU is restricted to transitioning between only three distinct belief states, implying a finite belief space within the *T* time slot horizon. Consequently, given a finite duration of *T* time slots, the belief space Γ constitutes a finite set.
- *States:* The discrete battery energy level of the SU at the start of time slot *t* is denoted by $b_{t}^{\prime }\in B≜\left\{0,\;1,\;\ldots ,\;b_{\max}\right\}$, where $b_{\max}$ signifies the SU’s maximum battery energy capacity. Consequently, the energy associated with each quantum is $\frac{B_{\max}}{b_{\max}}$ Joules. The continuous available battery energy of the SU is discretized into energy levels using the formula $b_{t}^{\prime }=\left\lfloor b_{t}\frac{b_{\max}}{B_{\max}}\right\rfloor$. The floor function applied here yields a lower bound on the AoI for the continuous system. Similarly, the continuous channel power gains are quantized into a finite number of levels according to the fading probability density function (PDF). These discrete levels of channel power gain are represented by $h_{t}^{\prime }\in H≜\left(0,\;1,\;2,\;\ldots ,\;h_{\max}\right)$ and $g_{t}^{\prime }\in G≜\left(0,\;1,\;2,\;\ldots ,\;g_{\max}\right)$, where $h_{\max}$ and $g_{\max}$ denote the peak channel power gain values for the SU-to-CBS link and the PU-to-SU link. There are fully observable states in each time slot, including the AoI state, the SU-to-CBS channel state, the PU-to-SU channel state, and the battery state, represented by $s_{t}≜\left(a_{t},\;h_{t}^{\prime },\;g_{t}^{\prime },\;b_{t}^{\prime }\right)$. It is important to note that the state space S≜(A×H×G×B) is finite. Furthermore, the PU spectrum state is partially observable and characterized by the belief $\varrho_{t}$. Consequently, for t=0,1,…,T−1, the entire system state is represented by $\left(s_{t},\varrho_{t}\right)$. Given the finite nature of both S and Γ, the SU can only encounter a limited number of possible system states $(s_{t},\varrho_{t})\in S\times \Gamma$.
- *Transition probabilities:* Given the current state $s_{t}=\left(a_{t},h_{t}^{\prime },g_{t}^{\prime },b_{t}^{\prime }\right)$ and action $x_{t}=\left(\theta_{t},\phi_{t}\right)$, the probability of transitioning to the next state $s_{t+1}=\left(a_{t+1},h_{t+1}^{\prime },g_{t+1}^{\prime },b_{t+1}^{\prime }\right)$ is expressed as $p_{x_{t}}\left(s_{t+1}|s_{t}\right)$. Since the harvested energy and the channel power gains are i.i.d, we have
  (10) $$p_{x_{t}}\left(s_{t+1}|s_{t}\right)=P\left(a_{t+1}|a_{t},\;x_{t}\right)P\left(b_{t+1}^{\prime }|b_{t}^{\prime },\;h_{t}^{\prime },\;g_{t}^{\prime },\;x_{t}\right)\times P\left(h_{t+1}^{\prime }\right)P\left(g_{t+1}^{\prime }\right),$$
  where
  (11) $$P\left(a_{t+1}|a_{t},\;x_{t}\right)=\left\{\begin{array}{c} 1,\;\;\;\;\mathrm{if}\;\;a_{t+1}=\left(1-\phi_{t}\right)a_{t}+1,\\ 0,\;\;\;\;\;\;\;\;\;\;\;\;\;\;\mathrm{otherwise}, \end{array}\right.$$
  (12) $$\begin{array}{c} P(b_{t+1}^{\prime }|b_{t}^{\prime },\;h_{t}^{\prime },\;g_{t}^{\prime },\;x_{t})=\\ \left\{\begin{array}{c} 1,\;\mathrm{if}\;\;\theta_{t}=0\;,\;b_{t+1}=\min\left\{\;b_{t}+E_{H,1}^{t},\;B_{\max}\right\},\\ 1,\;\mathrm{if}\;\;\theta_{t}=0\;,\;b_{t+1}=b_{t},\\ 1,\;\mathrm{if}\;\;\theta_{t}=1\;,\;\phi_{t}=0\;,\;b_{t+1}=\min\left\{b_{t}-\delta +E_{H,2}^{t},\;B_{\max}\right\},\\ 1,\;\mathrm{if}\;\;\theta_{t}=1\;,\;\phi_{t}=0\;,\;b_{t+1}=b_{t}-\delta ,\\ 1,\;\mathrm{if}\;\;\theta_{t}=1\;,\;\phi_{t}=1\;,\;b_{t+1}=b_{t}-\delta -E_{T}^{t},\\ 0,\;\mathrm{otherwise}. \end{array}\right. \end{array}$$
  Equation (12) means that battery state transition probability is 1 if the battery’s state changes according to the actual action; otherwise, it is 0.
- *Cost:* In the state $s_{t}$, the immediate cost is represented by $C(s_{t})$, where $C(s_{t})$ signifies the accumulated AoI at the time *t*. We then have
  (13) $$C\left(s_{t}\right)=a_{t},\;\;t=0,\;1,\;2,\;\ldots ,\;T-1.$$
- *Policy:* The policy π is defined as a sequence of deterministic decision rules $\left\{\nu_{0},\;\nu_{1},\;\ldots ,\;\nu_{T-1}\right\}$, where each rule, $\nu_{t}$, maps the system state $(s_{t},\varrho_{t})\in S\times \Gamma$ into an action, $x_{t}\in X$, i.e., $x_{t}=\nu_{t}\left(s_{t},\varrho_{t}\right)$. In this paper, let Π represent the set of all deterministic decision policies.

Given the SU’s initial state $s_{0}$ and belief $\varrho_{0}$, the finite-horizon AoI achieved by following the policy π is given by

(14) $${\bar{A}}^{\pi }\left(s_{0}.\varrho_{0}\right)=\frac{1}{T}E\left[\sum_{t=0}^{T-1}C\left(s_{t}\right)|s_{0},\;\varrho_{0}\right],$$

where the expectation is taken with respect to the policy π. Based on the above analysis, the problem of determining the optimal sensing and updating policy for minimizing the average AoI of the SU is equivalent to solving

(15) $$\min_{\pi \in \Pi }\;{\bar{A}}^{\pi }\left(s_{0},\varrho_{0}\right).$$

Equation (15) represents a finite-state MDP with total cost under a given *T*.

### 3.2. Dynamic Programming-Based POMDP Solution

To solve the finite-horizon total cost minimization problem given by Equation (15), we apply dynamic programming [40]. The state-value function, denoted by $V_{t}\left(s_{t},\varrho_{t}\right)$, is expressed as

(16) $$V_{t}\left(s_{t},\varrho_{t}\right)≜\min_{{\left\{x_{k}\right\}}_{k=t}^{T-1}}E\left[\sum_{k=t}^{T-1}C\left(s_{k}\right)|s_{t},\varrho_{t}\right].$$

It represents the minimum expected cost accumulated from the time slot *t* to T−1 given the state $\left(s_{t},\varrho_{t}\right)$. Therefore, the minimum AoI in Equation (15) is $A^{*}=V_{0}\left(s_{0},\varrho_{0}\right)/T$. Similarly, given $\left(s_{t},\varrho_{t}\right)$ and a sensing action, $\theta_{t}$, let $Q_{t}^{\theta_{t}}\left(s_{t},\varrho_{t}\right)$ represent the Q-function or action value function, which corresponds to the minimum expected cost for taking the sensing action $\theta_{t}$ in the state $\left(s_{t},\;\varrho_{t}\right)$. The Q-function consists of two parts: the immediate cost for taking action in the current state and the expected sum of the state-value functions from the next time slot. The finite-horizon MDP problem can be solved recursively via dynamic programming as follows. For t=0,1,…,T−1,

(17) $$V_{t}\left(s_{t},\varrho_{t}\right)=\min_{\theta_{t}\in \Gamma_{\theta }}Q_{t}^{\theta_{t}}\left(s_{t},\varrho_{t}\right),$$

where for t=T−1,

(18) $$Q_{T-1}^{0}\left(s_{T-1},\varrho_{T-1}\right)=C\left(s_{T-1}\right)+C\left(s_{T}\right),$$

(19) $$Q_{T-1}^{1}\left(s_{T-1},\varrho_{T-1}\right)=\left(1-\varrho_{T-1}\right)C\left(s_{T-1}\right)+\varrho_{T-1}\times \min_{\phi_{T-1}\in \Gamma_{\phi }}C\left(s_{T-1}\right)+C\left(s_{T}\right).$$

and for t=0,1,…,T−2,

(20) $$Q_{t}^{0}\left(s_{t},\varrho_{t}\right)=C\left(s_{t}\right)+\sum_{s_{t+1}}p_{00}\left(s_{t+1}|s_{t}\right)V_{t+1}\left(s_{t+1},\;\Lambda_{0}\left(\varrho_{t}\right)\right),$$

(21) $$Q_{t}^{1}\left(s_{t},\varrho_{t}\right)=\left(1-\varrho_{t}\right)Q_{t}^{1A}\left(s_{t},\varrho_{t}\right)+\varrho_{t}\min_{\phi_{t}\in \Gamma_{\phi }}Q_{t}^{1\phi_{t}}\left(s_{t},\varrho_{t}\right),$$

(22) $$Q_{t}^{1A}\left(s_{t},\varrho_{t}\right)=C\left(s_{t}\right)+\sum_{s_{t+1}}p_{10}\left(s_{t+1}|s_{t}\right)V_{t+1}\left(s_{t+1},\;\Lambda_{A}\left(\varrho_{t}\right)\right),$$

(23) $$Q_{t}^{10}\left(s_{t},\varrho_{t}\right)=C\left(s_{t}\right)+\sum_{s_{t+1}}p_{10}\left(s_{t+1}|s_{t}\right)V_{t+1}\left(s_{t+1},\;\Lambda_{I}\left(\varrho_{t}\right)\right),$$

(24) $$Q_{t}^{11}\left(s_{t},\varrho_{t}\right)=C\left(s_{t}\right)+\sum_{s_{t+1}}p_{11}\left(s_{t+1}|s_{t}\right)V_{t+1}\left(s_{t+1},\;\Lambda_{I}\left(\varrho_{t}\right)\right).$$

Specifically, when the sensing action $\theta_{t}=1$ is implemented and produces the result ${\hat{q}}_{t}=A$, the minimum expected cost is represented by $Q_{t}^{1A}\left(s_{t},\varrho_{t}\right)$ in Equation (22), i.e., $x_{t}=\left(1,0\right)$. Given the sensing action $\theta_{t}=1$ and the sensing result ${\hat{q}}_{t}=I$ in Equations (23) and (24), $Q_{t}^{10}\left(s_{t},\varrho_{t}\right)$ and $Q_{t}^{11}\left(s_{t},\varrho_{t}\right)$ denote the minimum expected costs associated with implementing the update actions $\phi_{t}=0$ and $\phi_{t}=1$, respectively. Then, by the recursion in (17)–(24), the optimal policies for sensing and update are obtained by

(25) $$\theta_{t}^{*}\left(s_{t},\varrho_{t}\right)\in \underset{\theta_{t}\in \Gamma_{\theta }}{\mathrm{argmin}}\;Q_{t}^{\theta_{t}}\left(s_{t},\varrho_{t}\right),$$

(26) $$\phi_{t}^{*}\left(s_{t},\varrho_{t}\right)\in \underset{\phi_{t}\in \Gamma_{\phi }}{\mathrm{argmin}}\;Q_{t}^{1\phi_{t}}\left(s_{t},\varrho_{t}\right).$$

## 4. System Model for RF Energy-Harvesting CRN with Multiple SUs

**Figure 2:**
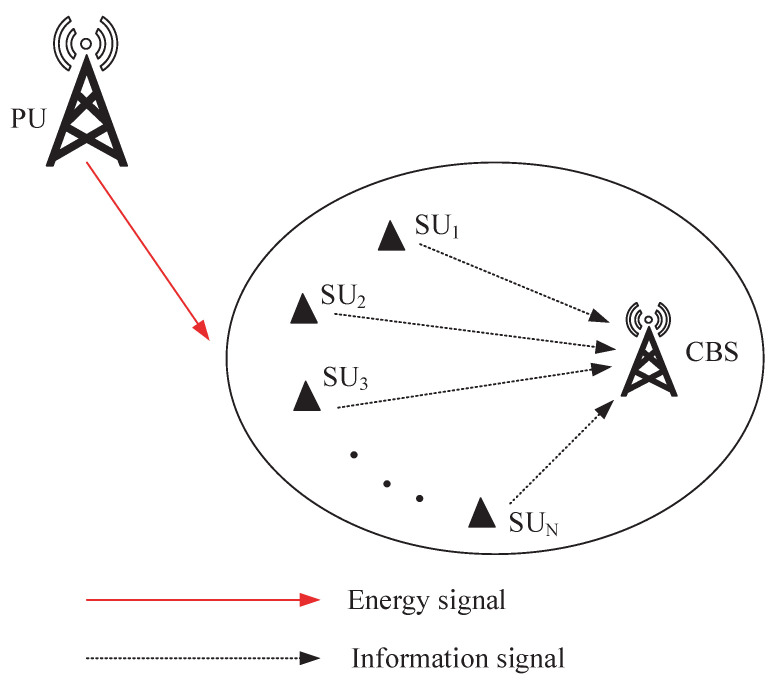
System model for multiple SUs.

We further study the long-term average weighted sum-AoI minimization in an RF energy-harvesting CRN with multiple SUs, as shown in Figure 2. During the time slot *t*, all SUs concurrently capture energy from the PU transmissions, or a single SU is designated for spectrum sensing while another (possibly the same or a different) SU is selected for accessing the available spectrum. Given the similarity to the RF energy-harvesting CRN with a single SU, we will not go into detail about the PU and belief model in this section, and we only provide a brief description of the SU model as follows.

### Secondary Users Model

The central controller (CC) makes the sensing and update decisions for the SUs and broadcasts the decision to them at the beginning of each time slot, *t*. The decision that none of the SUs sense the spectrum means that all the SUs capture energy from the PU transmissions in the entire time slot. The decision to sense the spectrum means deciding which SU is selected to sense the spectrum. If the selected SU is sensing the spectrum, other SUs are in the energy-harvesting state. We assume the perfect sensing outcome for the SUs. If the sensing result indicates the spectrum is occupied by the PU, all the SUs harvest energy in the rest time of this time slot. On the other hand, if the spectrum is sensed to be vacated by the PU, the CC needs to further decide whether to assign one SU to deliver the status update data pack and which one should do so. By making optimal decisions sequentially across time slots, the CC endeavors to minimize the long-term average of the weighted sum-AoI for the SUs. $x_{t}=\left(\theta_{t},\phi_{t}\right)$ denotes the decision taken in the time slot *t*, where $\theta_{t}\in \left\{0,\;1,\;\ldots ,\;N\right\}$ and $\phi_{t}\in \left\{0,\;1,\;\ldots ,\;N\right\}$. In particular, $\theta_{t}=0$ implies that all the SUs capture energy from the PU transmissions, while $\theta_{t}=1,\;2,\;\ldots N$ means which SU is selected to sense the spectrum. $\phi_{t}=0$ implies that no SU is designated to deliver the status update data packet, whereas $\phi_{t}=1,\;2,\;\ldots N$ means which SU is selected to deliver the status update data pack. Decisions are made based on the individual state of each SU and all the SUs’ statistical knowledge of the PU activity, discussed as follows.

*(1) Energy-harvesting model:* Energy harvesting can occur under two different scenarios: (1) at the beginning of the time slot, no sensing action is performed, and the PU remains active; (2) a sensing decision is made at the beginning of the time slot, and the sensing result indicates that the spectrum is busy. For the second case, all SUs start to harvest energy when the sensing action ends, and all non-sensing SUs harvest energy while the sensing SU is engaged in the sensing process. Let $g_{n,t}$ represent the channel power gain from the PU to the *n*-th SU. The energy captured by the *n*th SU is given by

(27) $$E_{H_{m,n}}^{t}=\tau \eta Pg_{n,t},\;\;t=0,\;1,\;\ldots ,\;T-1,\;m=1,\;2,$$

where the two different values of *m* corresponding to the two cases for energy harvesting described above. Let $\delta_{n}$ and $\tau_{s,n}$ denote the energy and time consumption for sensing, respectively, for the *n*-th SU. Additionally, let $E_{T,n}^{t}$ and $\tau_{t,n}$ represent the energy and time consumption for transmission in the time slot *t* for the *n*-th SU, respectively. According to Shannon’s formula, $E_{T,n}^{t}$ is expressed as

(28) $$E_{T,n}^{t}=\frac{\sigma^{2}\tau_{t,n}}{h_{t,n}}\left(2^{\frac{S}{\tau_{i}W}}-1\right),$$

where $h_{t,n}$ represents the channel power gain from the *n*-th SU to the CBS. The *n*-th SU possesses a battery capacity of $B_{\max,n}$ Joules.

The battery state in the time slot *t* for the *n*-th SU is denoted by $b_{t,n}$, which evolves as

(29) $$b_{t+1,n}=\min\left\{b_{t,n}+E_{H_{m,n}}^{t}-1\left(\theta_{t}=n\right)\delta_{n}-1\left(\phi_{t}=n\right)E_{T,n}^{t},\;B_{\max,n}\right\},\;\;\;\;\;\;\;t=0,\;1,\;2,\;\ldots ,\;T\;-\;1,$$

where 1(.) represents the indicator function. The energy causality constraint for the *n*th SU is satisfied by

(30) $$1\left(\theta_{t}=n\right)\delta_{n}+1\left(\phi_{t}=n\right)E_{T,n}^{t}\leq b_{t,n},\;\;t=0,\;1,\;\ldots ,\;T-1.$$

*(2) Age of information:* We denote AoI by $a_{t,n}$ in time slot *t* for the *n*th SU’s observed process *n*. We assume that the upper bound of $a_{t,n}$ is $A_{\max,n}$, which can be selected as an arbitrarily large value, i.e., $a_{t,n}\in A_{n}≜\left\{1,\;2,\;\ldots ,\;A_{\max,n}\right\}$. Note that when $a_{t,n}$ reaches $A_{\max,n}$, the information available at the CBS regarding the process *n* is considered excessively outdated, rendering further tracking unnecessary. The *n*th SU’s AoI evolves over time slots and is expressed as

(31) $$a_{t+1,n}=\left\{\begin{array}{c} 1,\;\;\;\;\;\;\mathrm{if}\;\;\phi_{t}=n,\\ a_{t,n}+1,\;\;\;\;\;\;\;\;\;\mathrm{otherwise}. \end{array}\right.$$

## 5. INFINITE POMDP FORMULATION for RF Energy-Harvesting CRN with Multiple SUs

### 5.1. POMDP Formulation

To achieve the minimization of the SUs’ long-term weighted sum-AoI, the optimal sensing and update decisions are modeled as a POMDP. The constituent elements of this POMDP are detailed below. Note that the policy description is not elaborated on here, and some other details are neglected. For a more detailed explanation, please refer to Section 3.

- *Actions:* The CC decides the sensing SU and the updating SU. The action implemented within each time slot is $x_{t}=\left(\theta_{t},\phi_{t}\right)\in X≜\left\{\left(0,0\right),\;\left(1,0\right),\;\left(1,1\right),\;\ldots ,\;\left(n,n\right):b_{t,n}\geq 1\left(\theta_{t}=n\right)\delta_{n}+1\left(\phi_{t}=n\right)E_{T,n}^{t}\right\}$, where $\theta_{t}\in \Gamma_{\theta }≜\left\{0,1,\ldots ,n:b_{t,n}\geq 1\left(\theta_{t}=n\right)\delta_{n}\right\}$ and $\phi_{t,n}\in \Gamma_{\phi }≜\left\{0,1,\ldots ,n:b_{t,n}\geq \delta_{n}+1\left(\phi_{t}=n\right)E_{T,n}^{t}\right\}$.
- *Observations and beliefs:* Let $\varrho_{t}^{\prime }\in R≜\left\{0,1,2,\ldots ,\varrho_{\max}\right\}$ denote the discrete belief level at the beginning of the time slot *t*, where $\varrho_{\max}$ represents the maximum belief level. In this case, the continuous belief $\varrho_{t}$ can be converted into the discrete belief level according to $\varrho_{t}^{\prime }=\left\lfloor \frac{\varrho_{t}}{1/\varrho_{\max}}\right\rfloor$.
- *State:* The discrete battery energy level of the *n*-th SU at the start of the time slot *t* is denoted by $b_{t,n}^{\prime }$, where $b_{t,n}^{\prime }$ belongs to the set $B_{n}≜\left\{0,\;1,\;\ldots ,\;b_{\max,n}\right\}$. Here, $b_{\max,n}$ represents the maximum energy storage capacity of the *n*-th SU’s battery. Thus, each energy quantum of the *n*-th SU’s battery corresponds to $\frac{B_{\max,n}}{b_{\max,n}}$ Joules. In this case, the *n*-th SU’s continuous battery energy can be converted into the discrete battery energy level state according to $b_{t,n}^{\prime }=\left\lfloor b_{t,n}\frac{b_{\max,n}}{B_{\max,n}}\right\rfloor$. Likewise, the continuous channel power gains for the links between the *n*-th SU and the CBS, and the PU and the *n*-th SU, are mapped to discrete levels, i.e., $h_{t,n}^{\prime }\in H_{n}≜\left(0,\;1,\;2,\;\ldots ,\;h_{\max,n}\right)$ and $g_{t,n}^{\prime }\in G_{n}≜\left(0,\;1,\;2,\;\ldots ,\;g_{\max,n}\right)$, where $h_{\max,n}$ and $g_{\max,n}$ signify the upper bounds of the channel power gain levels from the *n*-th SU to the CBS and from the PU to the *n*-th SU, respectively. The completely observable state of the *n*-th SU at any time slot, *t*, is composed of its AoI value, the channel condition between it and the CBS, the channel condition from the PU to it, and its residual battery energy. These are denoted by $s_{t,n}≜\left(a_{t,n},\;h_{t,n}^{\prime },\;g_{t,n}^{\prime },\;b_{t,n}^{\prime }\right)$. The state of all the SUs in the time slot *t* is represented by $s_{t}\in S={\left\{s_{t,n}\right\}}_{n\in N}$. Integrating the PU spectrum belief, the complete system state is denoted by $\left(s_{t},\varrho_{t}^{\prime }\right)$.
- *Transition probabilities:* For the *n*-th SU, the transition probability from the current state $s_{t,n}=\left(a_{t,n},h_{t,n}^{\prime },g_{t,n}^{\prime },b_{t,n}^{\prime }\right)$ to the next state $s_{t+1,n}=\left(a_{t+1,n},h_{t+1,n}^{\prime },g_{t+1,n}^{\prime },b_{t+1,n}^{\prime }\right)$ under the action $x_{t}=\left(\theta_{t},\phi_{t}\right)$ is given by
  (32) $$p_{x_{t}}\left(s_{t+1,n}|s_{t,n}\right)=P\left(a_{t+1,n}|a_{t,n},\;x_{t}\right)P\left(b_{t+1,n}^{\prime }|b_{t,n}^{\prime },\;h_{t,n}^{\prime },\;g_{t,n}^{\prime },\;x_{t}\right)\times P\left(h_{t+1,n}^{\prime }\right)P\left(g_{t+1,n}^{\prime }\right),$$
  where
  (33) $$P\left(a_{t+1,n}|a_{t,n},\;x_{t}\right)=\left\{\begin{array}{c} 1,\;\mathrm{if}\;\;a_{t+1,n}=\left(1-1\left(\phi_{t}=n\right)\right)a_{t,n}+1,\\ 0,\;\mathrm{otherwise}, \end{array}\right.$$
  (34) $$\begin{array}{c} P(b_{t+1,n}^{\prime }|b_{t,n}^{\prime },\;h_{t,n}^{\prime },\;g_{t,n}^{\prime },\;x_{t})=\\ \left\{\begin{array}{c} 1,\;\mathrm{if}\;\;\theta_{t}=0\;,\;b_{t+1,n}=\min\left\{b_{t,n}+E_{H_{1,n}}^{t},\;B_{\max,n}\right\},\\ 1,\;\mathrm{if}\;\;\theta_{t}=0\;,\;b_{t+1,n}=b_{t,n},\\ 1,\;\mathrm{if}\;\;\theta_{t}=n\;,\;\phi_{t}=0\;,\;b_{t+1,n}=\min\left\{b_{t,n}-\delta_{n}+E_{H_{2,n}}^{t},\;B_{\max,n}\right\},\\ 1,\;\mathrm{if}\;\;\theta_{t}\neq n\;,\;\phi_{t}=0\;,\;b_{t+1,n}=\min\left\{b_{t,n}+E_{H_{1,n}}^{t},\;B_{\max,n}\right\},\\ 1,\;\mathrm{if}\;\;\theta_{t}=n\;,\;\phi_{t}=0\;,\;b_{t+1,n}=b_{t,n}-\delta_{n},\\ 1,\;\mathrm{if}\;\;\theta_{t}=n\;,\;\phi_{t}=n\;,\;b_{t+1,n}=b_{t,n}-\delta_{n}-E_{T,n}^{t},\\ 1,\;\mathrm{if}\;\;\theta_{t}=n\;,\;\phi_{t}\neq n\;,\;b_{t+1,n}=b_{t,n}-\delta_{n},\\ 1,\;\mathrm{if}\;\;\theta_{t}\neq n\;,\;\phi_{t}=n\;,\;b_{t+1,n}=b_{t,n}-E_{T,n}^{t},\\ 1,\;\mathrm{if}\;\;\theta_{t}\neq n,\;\theta_{t}\neq 0,\;\phi_{t}\neq n,\;\phi_{t}\neq 0,\;b_{t+1,n}=b_{t,n},\\ 0,\;\mathrm{otherwise}. \end{array}\right. \end{array}$$
  Then, the overall transition probability is given by
  (35) $$P_{x_{t}}\left(s_{t}^{\prime }|s_{t}\right)=\prod_{n\in N}P_{x_{t}}\left(s_{t,n}^{\prime }|s_{t,n}\right).$$
- *Cost:* Let the immediate cost incurred in the time slot *t* in the state $s_{t}$ be denoted by $C(s_{t})$, quantifying the weighted sum-AoI at that specific time instant. Therefore, we obtain
  (36) $$C\left(s_{t}\right)=\sum_{n=1}^{N}\beta_{n}a_{t,n},\;\;t=0,\;1,\;2,\;\ldots ,\;T-1,$$
  where $\beta_{n}\geq 0$ and $\sum_{n=1}^{N}\beta_{n}=1$. Here, $\beta_{n}$ signifies a weighting parameter that modulates the impact of the physical process *n* as observed at the CBS.

Then, the optimal policy can be derived by solving the Bellman equations presented below [40].

(37) $${\bar{A}}^{*}+V_{t}\left(s_{t},\varrho_{t}^{\prime }\right)=\min_{x_{t}\in A\left(s_{t},\varrho_{t}^{\prime }\right)}Q_{t}\left(s_{t},\varrho_{t}^{\prime },x_{t}\right),\;\forall \left(s_{t},\varrho_{t}^{\prime }\right)\in S\times R,$$

where ${\bar{A}}^{*}$ signifies the optimal average AoI which does not depend on the initial state $\left(s_{0},\varrho_{0}^{\prime }\right)$, $V_{t}\left(s_{t},\varrho_{t}^{\prime }\right)$ is the state-value function, $A(s_{t},\varrho_{t}^{\prime })$ is the action taken in the state $\left(s_{t},\varrho_{t}^{\prime }\right)$, and $Q_{t}\left(s_{t},\varrho_{t}^{\prime },x_{t}\right)$ is the Q-function, which is given by

(38) $$Q_{t}\left(s_{t},\varrho_{t}^{\prime },x_{t}\right)=\sum_{n=1}^{N}\beta_{n}a_{t,n}+\sum_{s_{t+1}\in S}P_{x_{t}}\left(s_{t+1}|s_{t}\right)V_{t}\left(s_{t+1},\varrho_{t+1}^{\prime }\right).$$

Therefore, given all the SUs’ states, $s_{t}$, and the belief $\varrho_{t}^{\prime }$, the optimal action is given by

(39) $$\pi^{*}\left(s_{t},\varrho_{t}^{\prime }\right)=\arg\;\min_{x_{t}\in A\left(s_{t},\varrho_{t}^{\prime }\right)}Q_{t}\left(s_{t},\varrho_{t}^{\prime },x_{t}\right).$$

Given the intractably large state space that grows exponentially with the number of SU nodes and the granularity of state discretization, we propose a DRL approach in the subsequent subsection to determine the optimal policy.

### 5.2. DRL-Based POMDP Solution

The most common DRL algorithm is Q-learning, which has been widely applied in network resource optimization. In the Q-learning algorithm, the update step for the Q-function value of the current state occurs at the beginning of each time slot, based on the action taken and the resulting next state [41]. Specifically, the Q-learning algorithm’s update mechanism for the present problem, initiated at the onset of the time slot t+1, is given by

(40) $$\begin{array}{cc} Q_{t+1}\left(s_{t},\varrho_{t}^{\prime },x_{t}\right)= & Q_{t}\left(s_{t},\varrho_{t}^{\prime },x_{t}\right)+\alpha \left(t\right)(C\left(s_{t}\right)\\ \; & +\min_{\bar{x}\in A\left(s_{t+1},\varrho_{t+1}^{\prime }\right)}Q_{t}\left(s_{t+1},\varrho_{t+1}^{\prime },\bar{x}\right)\\ \; & -\min_{\bar{x}\in A\left(\bar{s},\;\bar{\varrho }\right)}Q_{t}\left(\bar{s},\;{\bar{\varrho }}^{\prime },\bar{x}\right)-Q_{t}\left(s_{t},\varrho_{t}^{\prime },x_{t}\right)), \end{array}$$

where α(t) denotes the learning rate parameter in the time slot *t*, $A(s_{t+1},\;\varrho_{t+1}^{\prime })\in X$ and $A(\bar{s},\;{\bar{\varrho }}^{\prime })\in X$ are the actions taken in the states $\left(s_{t+1},\varrho_{t+1}^{\prime }\right)$ and $\left(\bar{s},{\bar{\varrho }}^{\prime }\right)$, and $\left(\bar{s},{\bar{\varrho }}^{\prime }\right)$ denotes the time-invariant reference state, a constant value across all iterations that can be defined arbitrarily. Based on (40), the system invariably leverages the learning process by choosing the action that yields the minimum Q-function value for the current state. However, for the algorithm to achieve convergence, the exhaustive exploration of the state–action space is imperative. Consequently, the ϵ-greedy policy is adopted, where a randomized action is chosen in the present state with a probability 0<ϵ<1. This policy allows the system to explore the environment rather than solely exploiting the learned knowledge.

Employing the Q-learning algorithm in isolation to determine the optimal policy proves efficacious when the cardinality of the system’s state space is limited. However, in scenarios involving an exceedingly large number of states, as encountered in our problem, maintaining the Q-function values for all state–action combinations becomes infeasible, and ensuring comprehensive visitation of all such pairs is also challenging, thus impeding convergence. To overcome this challenge, the DQN approach is utilized. While DQN retains the fundamental learning steps of Q-learning, it employs a DNN, Q(s, $\varrho^{\prime }$, x|ξ), to approximate the Q-function, with ξ signifying the vector of the DNN’s parameters. To ensure that the stored Q-function approximated by the DNN is as close as possible to the optimal Q-function, the optimal values of ξ must be found. To this end, a loss function for any tuple ($s_{t}$, $\varrho_{t}^{\prime }$, $x_{t}$, $C(s_{t})$, $s_{t+1}$, $\varrho_{t+1}^{\prime }$) is defined as

(41) $$L\left(\xi_{t+1}\right)={\left(C\left(s_{t}\right)+\min_{\bar{x}\in A\left(s_{t+1},\;\varrho_{t+1}^{\prime }\right)}Q_{t}\left(s_{t+1},\;\varrho_{t+1}^{\prime },\;\bar{x}|\xi_{t}\right)-\min_{\bar{x}\in A\left(\bar{s},\;{\bar{\varrho }}^{\prime }\right)}Q_{t}\left(\bar{s},\;{\bar{\varrho }}^{\prime },\;\bar{x}|\xi_{t}\right)-Q_{t}\left(s_{t},\varrho_{t}^{\prime },x_{t}|\xi_{t+1}\right)\right)}^{2}.$$

Moreover, a replay memory is leveraged to store historical experiences, with each experience comprising the current state, the action performed, the immediate cost, and and the resultant next state. Following each time slot, a random batch of a finite size of past experiences is sampled from the replay memory, and the gradient of the DNN’s weights is computed as

(42) $$\begin{array}{cc} \Delta_{\xi_{t+1}}L\left(\xi_{t+1}\right)= & (C\left(s_{t}\right)+\min_{\bar{x}\in A\left(s_{t+1},\;\varrho_{t+1}^{\prime }\right)}Q_{t}\left(s_{t+1},\;\varrho_{t+1}^{\prime },\;\bar{x}|\xi_{t}\right)\\ \; & -\min_{\bar{x}\in A\left(\bar{s},\;{\bar{\varrho }}^{\prime }\right)}Q_{t}\left(\bar{s},\;{\bar{\varrho }}^{\prime },\;\bar{x}|\xi_{t}\right)-Q_{t}\left(s_{t},\varrho_{t}^{\prime },x_{t}|\xi_{t+1}\right))\\ \; & \times \Delta_{\xi_{t+1}}Q_{t}\left(s_{t},\;\varrho_{t}^{\prime },\;x_{t}|\xi_{t+1}\right). \end{array}$$

Then, this loss function is used to train the weights of the DNN.

Due to the partially observable of the PU spectrum, our problem cannot be solved by the traditional DQN. The actions generated by the DQN must belong to the environment’s action space, and the next state is determined by the environment dynamics. On the one hand, since our input is the belief of PU spectrum availability, the DQN network may generate an infeasible action. In such cases, we need to correct the DQN’s output action based on the actual sensed state of the PU spectrum. On the other hand, in the implementation of the DQN algorithm, we correct the PU spectrum state under the current state based on the action output from the DQN network in each time slot. As the DQN network continues to train, it will be able to predict the PU spectrum status more accurately. For example, for the action $x_{t}=\left(1,\;2\right)$, if the PU is in the active state, the second SU cannot perform the update. Thus, we need to make some adjustments to the traditional DQN to adapt it to our problem. When the sensing action ends, we correct the pre-selected action according to the real state of the PU spectrum. For the action $x_{t}=\left(1,\;2\right)$ just mentioned, first let first SU sense the spectrum. Given the sensing result, we decide whether to adjust the pre-selected action $x_{t}=\left(1,\;2\right)$. If the sensing result is that the PU is in the idle state, the second SU delivers the status update data pack during the remaining duration of this time slot. Otherwise, this pre-selected action is corrected to another one; that is, all the SUs capture energy during the remaining duration of this time slot, i.e., the corresponding real action is ${\hat{x}}_{t}=\left(1,\;0\right)$.

Overall, there are two cases in which the pre-selected actions are corrected: (1) the pre-selected action is related to the vacant PU spectrum, but the real state of this is busy; (2) the pre-selected action is related to the active PU spectrum, but the real state of this is idle.

For the first case, after performing sensing action, we correct the pre-selected action to another one; that is, all the SUs harvest energy in the rest time of this time slot. For the second case, after performing sensing action, we correct the pre-selected action to select the SU with the highest weighted to perform the update in the rest time of this time slot. Thus, the real action maybe the pre-selected one or another one. Specifically, when the pre-selected action does not meet the spectrum constraint, we give the pre-selected action a penalty, *p*, instead of the immediate cost $C(s_{t})$ to avoid it being chosen, and we store the pre-selected action together with its corresponding real action as experiences in the replay memory. Similarly, when the real action does not meet the energy causality constraint, in addition to giving a penalty, *p*, instead of the immediate cost $C(s_{t})$, the pre-selected action is reset to $x_{t}^{\prime }=\left(0,0\right)$ to avoid it being chosen. And we store the real action before reset together with the real action after reset as experiences in the replay memory.

Through simulations, we observe that as the number of training cycles increases, the action and the steady-state probability of the PU spectrum state gradually become consistent, and the actions that violate energy causality constraint can almost avoid being chosen. In particular, the new DQN approach we propose is adapted to the POMDP problem with the partially observable state modeled as a Markov chain. The proposed novel DQN approach’s algorithmic steps are summarized in Algorithm 1. Here, lines 13 and 17 specify the algorithmic operations required when the DQN generates an infeasible action (i.e., when the battery level falls below zero). Lines 19 to 21 indicate the operations the algorithm performs when the pre-selected action and the actual action do not match. Lines 22 to 23 indicate the operations the algorithm performs when the pre-selected action and the actual action are equal. Lines 26 to 27 indicate that a batch of samples is randomly drawn from the replay memory for training the DQN network. Although the use of the DQN helps address complexity, the proposed solution may still face scalability challenges as network size and environmental dynamics grow [42,43].   
**Algorithm 1:** The new DQN for average weighted sum-AoI minimization
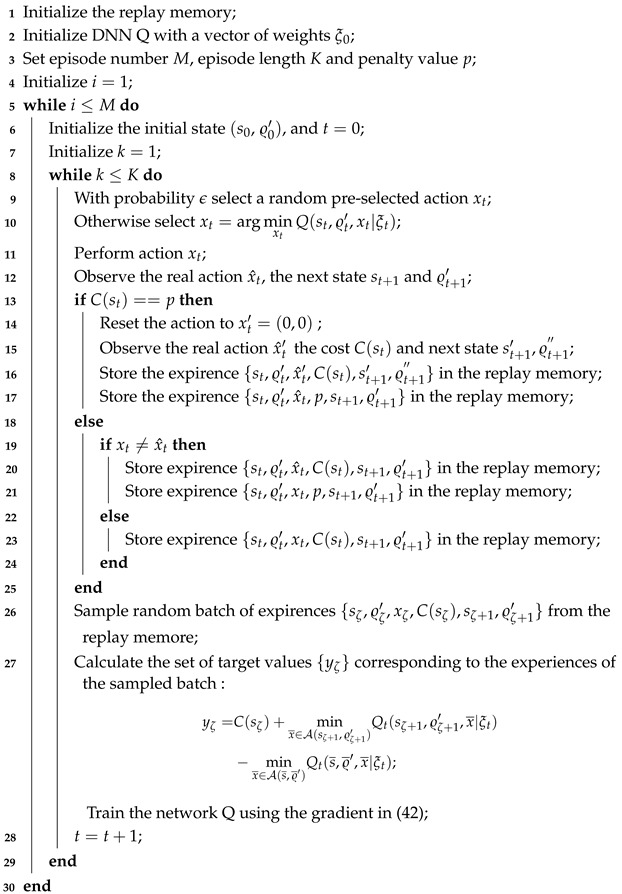


## 6. Numerical Results and Discussions

### 6.1. One SU’s Finite-Horizon AoI Evaluation

This section illustrates the performance evaluation of a specific scenario involving an SU via numerical results. The state transition probabilities of the PU are set as $p_{ii}=0.8$ and $p_{ai}=0.5$. The channel power gains between the SU and the CBS, and between the PU and the SU, are formulated as $h=\Upsilon \Psi^{2}d_{1}^{-\kappa }$ and $g=\Upsilon \Psi^{2}d_{2}^{-\kappa }$ [44]. Here, $d_{1}$ and $d_{2}$ are the distances from the SU to the CBS and from the PU to the SU, respectively. Υ signifies the signal power gain at a reference distance of one meter. Ψ∼exp(1) follows an exponential distribution with a mean of 1, representing the small-scale fading gain. $d_{1}^{-\kappa }$ and $d_{2}^{-\kappa }$ represent the conventional power-law path loss with the path loss exponent −κ. The proposed policy is compared to the myopic policy [31], a common benchmark. Under the myopic policy, provided that the SU possesses adequate energy for spectrum sensing, it undertakes this action; conversely, it harvests energy from the PU’s transmissions. When spectrum sensing indicates that the channel is occupied, the SU proceeds to harvest energy for the remainder of the time slot; otherwise, it transmits the status update data packet, contingent upon the residual energy being sufficient for an update. The simulation parameter values used in the simulations are shown in Table 1.

**Figure 3:**
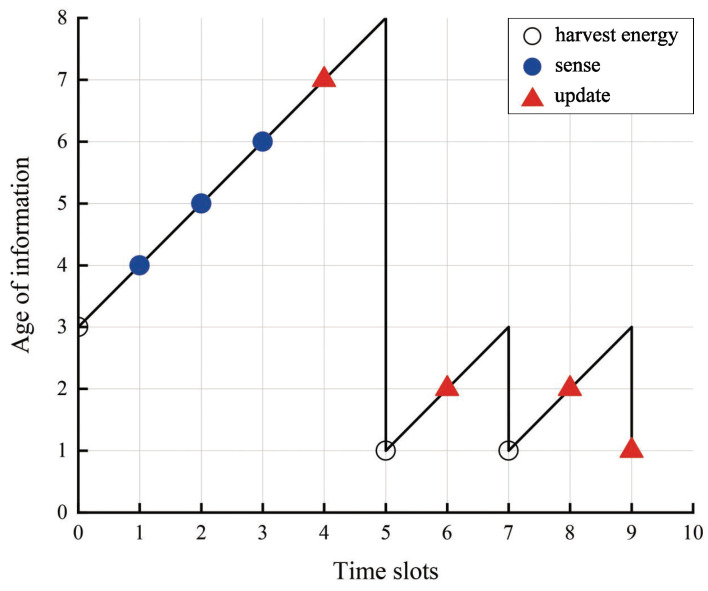
One sample path of AoI by the optimal policy.

Figure 3 illustrates a representative trajectory of the AoI achieved by employing the optimal policy. The PU-SU link distance is set to 5 m, and the SU-CBS link distance is configured at 25 m. The transmission power of the PU is set to 35 dBm, the battery has a capacity of 0.5 mJoules, and performing the sensing action requires an energy expenditure of one quantum. The corresponding system states and actions are listed in Table 2. The plot visually demonstrates the evolving trend of AoI across the time slots. Furthermore, it is observed that despite having adequate energy resources, the SU does not engage in spectrum sensing, which underscores the foresight inherent in our proposed strategy when compared to the myopic policy.

**Figure 4:**
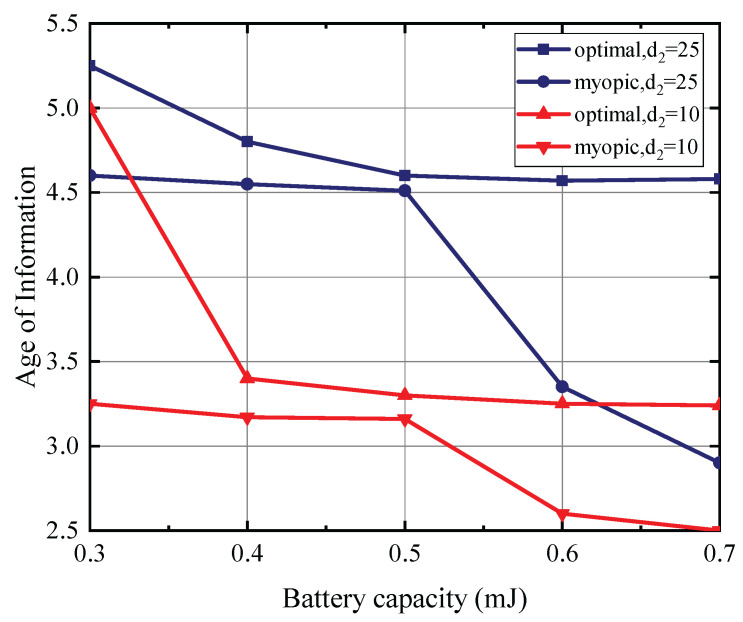
The batterry capacity versus AoI for T=10.

Figure 4 presents the relationship between battery capacity and AoI, with the PU’s transmit power set at 35 dBm, the status update data packet size at 15 Mbits, and the energy expenditure for sensing fixed at one energy quantum, based on a maximum battery capacity of 0.5 mJoules. The SU is located 5 m away from the PU. The presented results distinctly evidence the advantageous nature of the optimal policy over the myopic policy. We note that the average AoI exhibits a decreasing trend with an increase in battery capacity and a reduction in the distance between the SU and the CBS. This is attributed to the capacity of a larger battery to accommodate greater energy reserves, coupled with the fact that a diminished distance between the SU and the CBS curtails the energy expenditure necessary for the transmission of the status update data packet. Consequently, this enhances the likelihood of the SU possessing sufficient energy for transmitting the status update data packet, thereby leading to a reduction in its observed AoI.

**Figure 5:**
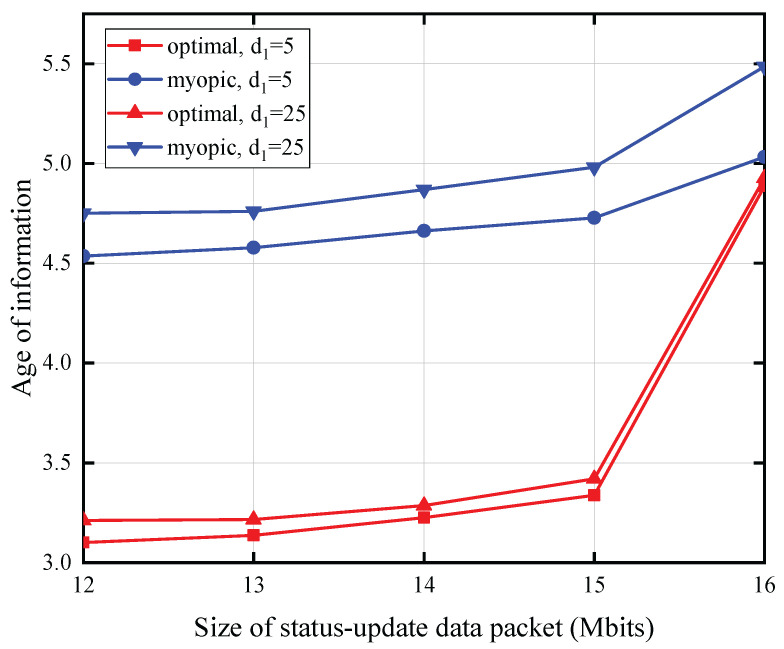
The size of status update data packet versus AoI for T=10.

Figure 5 illustrates the correlation between the status update data packet size and AoI, with the PU’s transmit power set at 35 dBm, the battery capacity at 0.2 mJoules, the energy expenditure for sensing at 0.125 mJoules, and the distance from the SU to the CBS fixed at 25 m. We note that the AoI exhibits an increasing trend with an increase in the size of the status update data packet and the distance between the PU and SU. This phenomenon arises from the fact that transmitting larger status update data packets necessitates greater energy expenditure, and an increased separation between the PU and SU leads to a reduction in the energy harvested by the SU. Consequently, the probability of the SU possessing sufficient energy for update packet transmission diminishes, resulting in an elevated AoI for the observed process.

**Figure 6:**
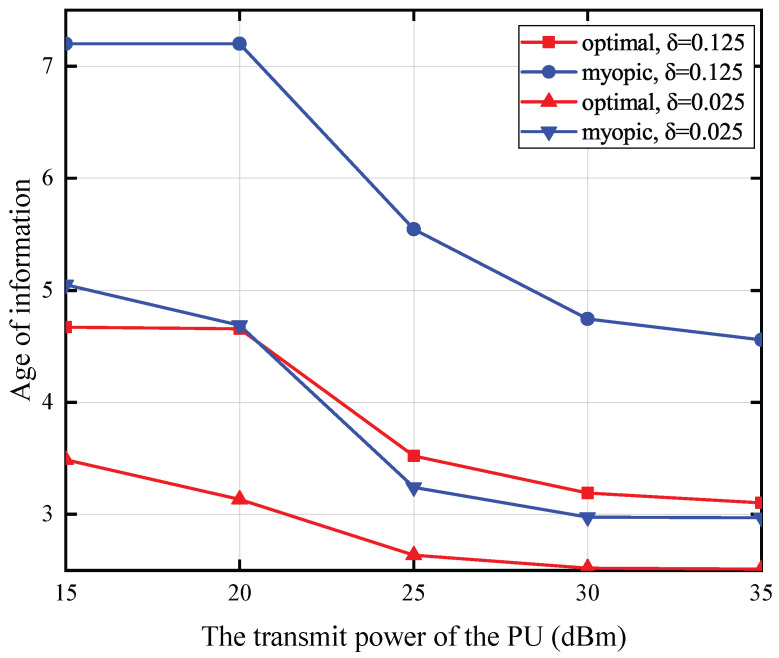
The transmit power of the PU versus AoI for T=10.

Figure 6 illustrates the correlation between the PU’s transmission power and the AoI under specific conditions: a battery capacity of 0.2 mJoules, a status update packet size of 15 Mbits, a PU-to-SU distance of 5 m, and an SU-to-CBS distance of 25 m. The data reveals an inverse relationship between the PU’s transmit power and the average AoI. specifically, higher transmit power correlates with a lower average AoI. Furthermore, a reduction in energy expenditure for the sensing operation is also associated with a decrease in the average AoI. The rationale behind this observation lies in the fact that a higher transmit power from the PU enhances the amount of energy that can be harvested and stored by the battery. Concurrently, lower energy expenditure for sensing activities preserves a greater energy reserve in the battery following the sensing phase. As a result, the probability of the SU possessing adequate energy to transmit the status update is elevated, which in turn leads to a decrease in the AoI of the monitored process. Moreover, the data reveals that under conditions of elevated energy expenditure for sensing, the optimal policy exhibits a considerably more pronounced performance advantage over the myopic policy when contrasted with scenarios involving lower sensing energy consumption. This disparity arises because the myopic policy, when confronted with high sensing energy demands and primary user spectrum occupancy, tends to expend more energy on superfluous sensing operations. Conversely, the optimal policy strategically manages the spectrum sensing decision, thereby mitigating energy wastage on redundant sensing activities.

### 6.2. Multiple-SU Infinite-Horizon AoI Evaluation

In this section, we evaluate the performance of the multiple-SU scenario through numerical results. We compare the proposed DQN approach with the myopic policy. For the myopic policy, we first sort the SUs in descending order of their weight. Then, we assign the first SU to sense the spectrum if it has enough energy. Otherwise, we assign the second highest-weighted SU to sense the spectrum, and so on. If the energy of each SU is insufficient to perform sensing, all SUs harvest energy from the PU transmissions. If the sensing result indicates that the spectrum is occupied, all SUs begin harvesting energy once the sensing action concludes. Upon determining that the spectrum is unoccupied, the SU with the highest-weighted and sufficient energy reserves is selected to transmit the status update data packet. If it does not, the second-highest weighted SU is assigned, and so on. In the simulations, unless otherwise specified, the parameter settings remained consistent with the scenario where only one SU is involved. Specifically, there were three SUs, each with a weight of 1/3. There is no fixed rule that dictates the exact number of SUs. The ideal quantity often depends on network scale, the available spectrum, and interference management. In many research studies and simulations, using three SUs is quite common. It is a good balance, simple enough to model and analyze yet complex enough to demonstrate interactions and potential interference scenarios among different users. The value of $\beta_{n}$ signifies that each SU’s observed physical process holds equal importance for the CBS. The discrete power gain for the channels connecting the PU to each SU, and likewise from each SU to the CBS, are uniformly configured at a value of 5. The upper limit for the AoI of each SU’s monitored process is set at 10, and each SU’s discrete battery energy level is initialized to 10 units. Furthermore, the battery capacity of each SU, the distance between the PU and each SU, and the distance between each SU and the CBS are all considered uniform across the system.

**Figure 7:**
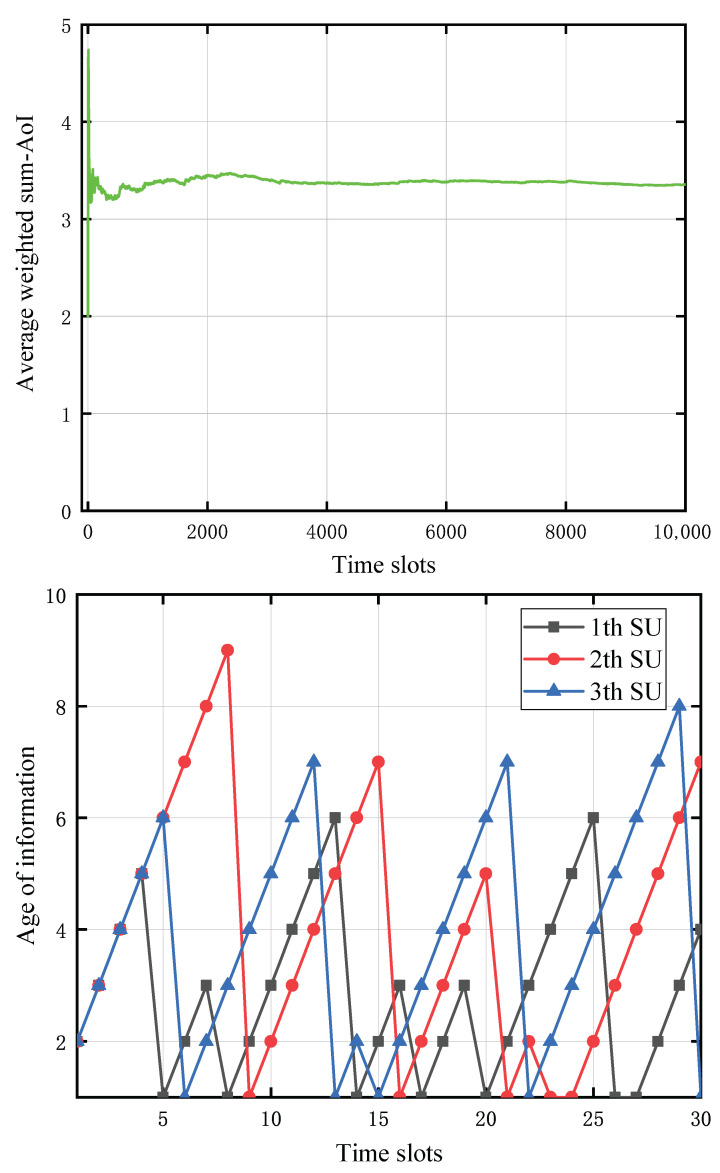
The changing trend of the average weighted sum-AoI and each SU’s AoI versus the time slots, respectively.

Figure 7 depict the temporal evolution of the average weighted sum-AoI and the individual AoI of each SU, respectively, across successive time slots. Under the conditions examined, the PU’s transmission power is set to 35 dBm, the status update packet size is 15 Mbits, each SU’s battery capacity is 0.5 mJoules, the distance from each SU to the CBS is 10 m, and the distance from the PU to each SU is 5 m. Each spectrum sensing operation incurs an energy expenditure of one quantum unit. From Figure 7, it is observed that the average weighted sum-AoI gradually stabilizes, which demonstrates the effectiveness of the proposed DQN approach. In order to clearly show the changing trend of the each SU’s AoI with the time slots, we only plot the first 30 time slots in Figure 7. We can observe that although each SU’s weight is set to be the same value, the peak AoI of the first SU is lower than the other two SUs. The AoI of the second and third SUs fluctuate more than that of the first SU.

**Figure 8:**
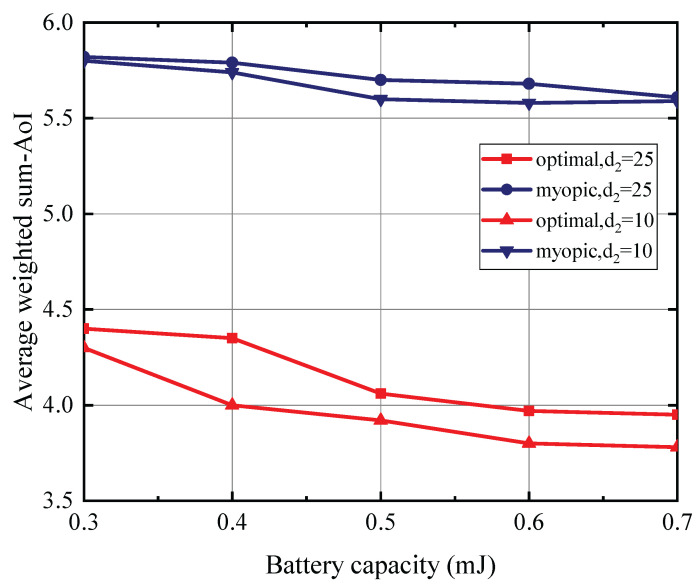
The battery capacity versus average weighted sum-AoI for *T* = 10,000.

Figure 8 illustrates the relationship between the battery capacity of each sSU and the resulting average weighted sum-AoI, given a PU transmit power of 35 dBm, a status update data packet size of 15 Mbits, and a distance of 5 m between the PU and each SU. The energy cost associated with each sensing operation is one quantum unit, determined with reference to each SU’s battery capacity of 0.5 mJoules. It can be observed that the proposed DQN approach significantly outperforms the myopic policy. Consistently with the findings observed in the single-SU scenario, a reduction in the distance between each SU and the CBS, as well as an increase in the battery capacity of each SU, leads to a decrease in the average weighted sum-AoI.

**Figure 9:**
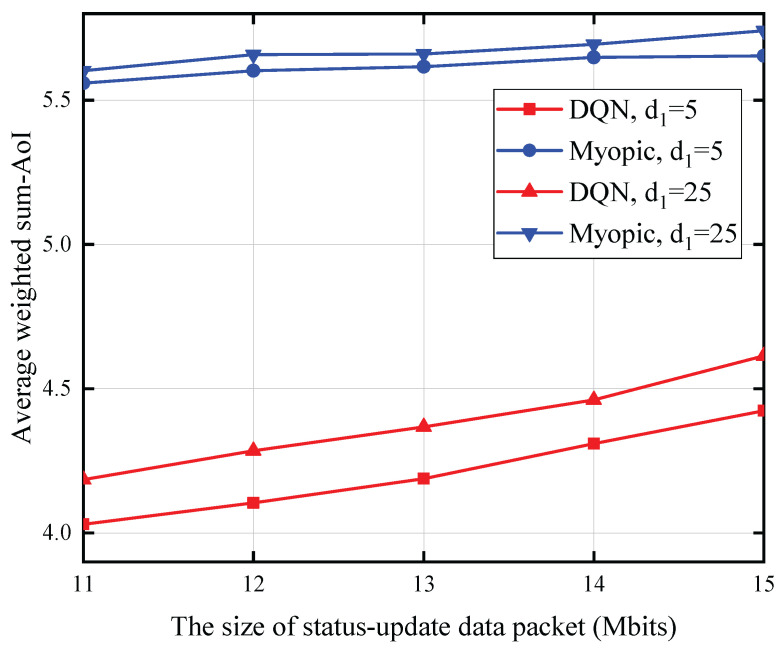
The status update date pack versus average weighted sum-AoI for *T* = 10,000.

Figure 9 depicts the correlation between the status update data packet size and the average weighted sum-AoI, under the conditions of a 15 dBm PU transmit power, a 0.5 mJoules battery capacity for each SU, a sensing energy consumption of one quantum unit, and a 20-m distance from each SU to the CBS. The data indicates a positive correlation between the average weighted sum-AoI and both the size of the status update data packet and the distance separating the PU from each SU.

**Figure 10:**
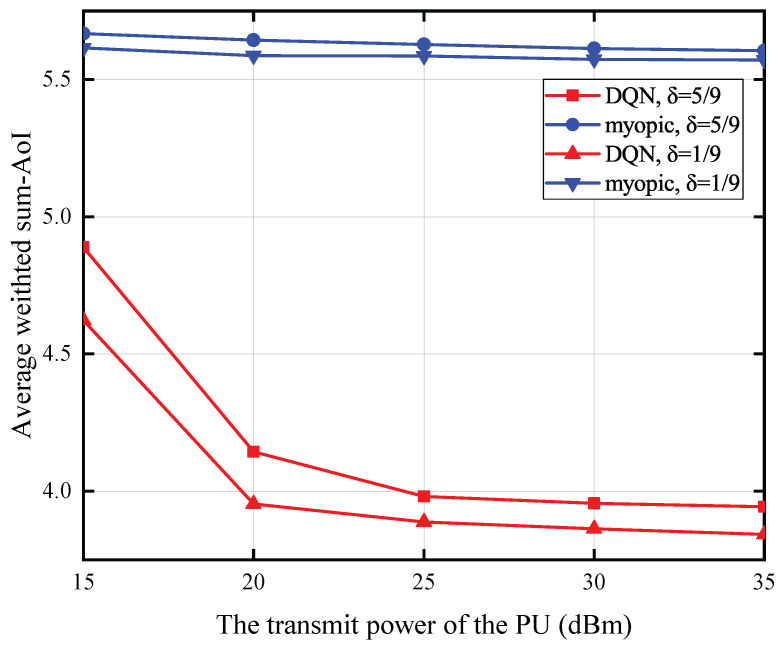
The transmit power of the PU versus average weighted sum-AoI for *T* = 10,000.

Figure 10 illustrates the connection between the PU’s transmission power and the average weighted sum-AoI, given that each SU has a battery capacity of 0.5 mJoules, the status update data packet size is 14 Mbits, the distance from the PU to each SU is 5 m, and the distance from each SU to the CBS is 25 m. The findings reveal an inverse relationship between the PU’s transmit power and the average weighted sum-AoI. Similarly, a reduction in the energy expended for sensing operations is associated with a lower average weighted sum-AoI.

## 7. Conclusions

In this paper, we first investigate a single RF energy-harvesting SU with the goal of AoI minimization. We adopt a POMDP to formulate the average AoI minimization problem, subject to energy causality and spectrum constraints. Then, dynamic programming is used to find the optimal decisions regarding energy harvesting, spectrum sensing, and information updating for AoI minimization. Furthermore, we extend our study to multiple RF energy-harvesting SUs, aiming to minimize the long-term average weighted sum-AoI. This problem is also formulated using the POMDP, and we propose an improved DQN to solve it. The numerical outcomes underscore the influence of various system parameters on overall performance and clearly demonstrate the substantial performance gains achieved by the proposed policies in comparison to myopic approaches. For future work, we will investigate alternative DRL approaches for CRNs, with special attention to imperfect sensing.

## Figures and Tables

**Table 1:** Simulation parameter values.

Simulation Parameter	Value
*W*	1 MHz
$\sigma^{2}$	−95 dBm
η	0.5
κ	2
Υ	0.2
$\tau_{s}$	0.2 s
$A_{\max}$	13
$h_{\max}$	10
$g_{\max}$	10
$\varrho_{0}$	$p_{ii}$
$b_{\max}$	5

**Table 2:** State–action pairs per time slot.

t	a	b	g	h	ϱ	Action
0	3	2	3	0	0.8	(0,0)
1	4	4	2	7	0.5	(1,0)
2	5	4	6	7	0.5	(1,0)
3	6	4	7	4	0.5	(1,0)
4	7	4	6	5	0.5	(1,1)
5	1	2	3	2	0.8	(0,0)
6	2	2	6	7	0.8	(1,1)
7	1	0	7	7	0.8	(0,0)
8	2	4	3	7	0.5	(1,1)
9	1	2	3	7	0.8	(1,1)

## Data Availability

Data are contained within the article.
